# 2-Chloro-3-[(2-oxo-2*H*-chromen-6-yl)amino]­naphthalene-1,4-dione

**DOI:** 10.1107/S1600536813019922

**Published:** 2013-07-27

**Authors:** Mikaelly O. B. Sousa, Gleiciani Q. Silveira, Javier A. G. Gomez

**Affiliations:** a Departamento de Química Inorgânica, Universidade Federal Fluminense, Niterói, CEP 24-020-140, Rio de Janeiro, Brazil

## Abstract

In the title compound, C_19_H_10_ClNO_4_, the dihedral angle between the naphtho­quinone and coumarin rings is 48.99 (6)°. In the crystal, mol­ecules are linked by strong N—H⋯O hydrogen bonds into chains with graph-set motif *C*(6) along [101]. The packing also features π–π stacking inter­actions between naphtho­quinone and coumarin rings [centroid-to-centroid distances = 3.7679 (12) and 3.6180 (13) Å].

## Related literature

For related compounds see: Rózsa *et al.* (1989 ▶); Ito *et al.* (1993 ▶); Ishikawa *et al.* (1995 ▶); Padwal *et al.* (2011 ▶). For reference structural data, see: Ibis & Deniz (2012 ▶); Resende & Gomez (2012 ▶). For graph-set notation of hydrogen bonds, see: Bernstein *et al.* (1995 ▶).
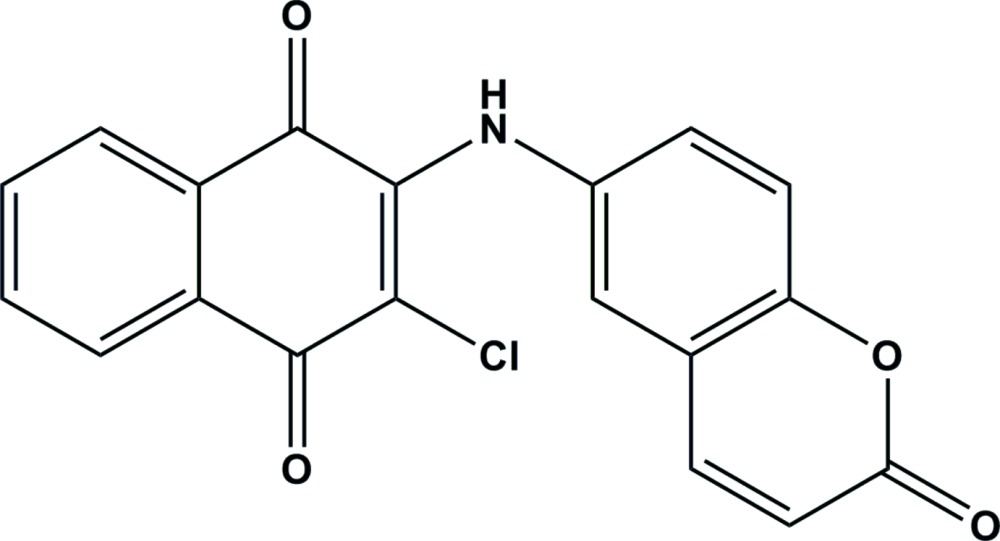


## Experimental

### 

#### Crystal data

C_19_H_10_ClNO_4_
*M* *_r_* = 351.73
Monoclinic, 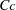

*a* = 10.9371 (5) Å
*b* = 10.4462 (5) Å
*c* = 13.5104 (7) Å
β = 108.533 (5)°
*V* = 1463.53 (12) Å^3^
*Z* = 4
Mo *K*α radiation
μ = 0.29 mm^−1^
*T* = 150 K
0.23 × 0.13 × 0.07 mm

#### Data collection

Oxford Xcalibur Gemini Ultra diffractometer with Atlas detector
Absorption correction: multi-scan (*CrysAlis PRO*; Agilent, 2011 ▶) *T*_min_ = 0.947, *T*_max_ = 1
15281 measured reflections
3527 independent reflections
2714 reflections with *I* > 2σ(*I*)
*R*_int_ = 0.055

#### Refinement

*R*[*F* ^2^ > 2σ(*F* ^2^)] = 0.036
*wR*(*F* ^2^) = 0.065
*S* = 0.91
3527 reflections
226 parameters
2 restraints
H-atom parameters constrained
Δρ_max_ = 0.23 e Å^−3^
Δρ_min_ = −0.21 e Å^−3^
Absolute structure: Flack (1983 ▶), 1217 Friedel pairs
Absolute structure parameter: −0.07 (5)

### 

Data collection: *CrysAlis PRO* (Agilent, 2011 ▶); cell refinement: *CrysAlis PRO*; data reduction: *CrysAlis PRO*; program(s) used to solve structure: *SHELXS97* (Sheldrick, 2008 ▶); program(s) used to refine structure: *SHELXL97* (Sheldrick, 2008 ▶); molecular graphics: *ORTEP-3 for Windows* (Farrugia, 2012 ▶); software used to prepare material for publication: *WinGX* (Farrugia, 2012 ▶).

## Supplementary Material

Crystal structure: contains datablock(s) global, I. DOI: 10.1107/S1600536813019922/bx2446sup1.cif

Structure factors: contains datablock(s) I. DOI: 10.1107/S1600536813019922/bx2446Isup2.hkl

Supplementary material file. DOI: 10.1107/S1600536813019922/bx2446Isup3.cml

Additional supplementary materials:  crystallographic information; 3D view; checkCIF report

## Figures and Tables

**Table 1:** Hydrogen-bond geometry (Å, °)

*D*—H⋯*A*	*D*—H	H⋯*A*	*D*⋯*A*	*D*—H⋯*A*
N1—H1⋯O2^i^	0.86	2.21	3.015 (2)	157

Symmetry code: (i) 
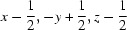
.

**Fig. 1.**
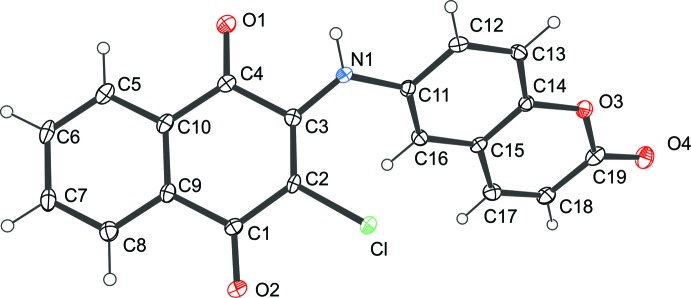
ORTEP representation (Farrugia, 2012) of the molecular structure of compound (I) with the numbering and displacement ellipsoids (at 30% probability level).

**Fig. 2.**
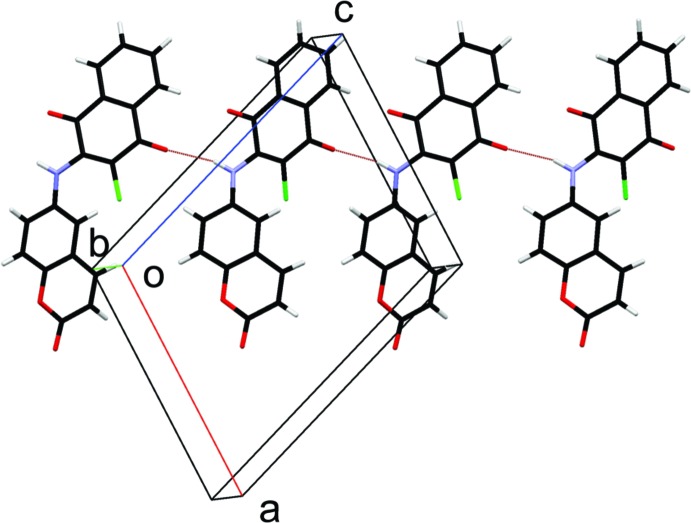
Packing diagram of (I), showing the formation of the C(6) chain along [101]. Hydrogen-bonds are shown by dashed lines.

## supplementary crystallographic information

### Comment

There are very few examples in the literature of coumarin–naphthoquinone conjugates, most of them (direct C—C bond) are from natural sources (Rózsa *et al.*, 1989; Ito *et al.*, 1993; Ishikawa *et al.*, 1995; Padwal *et al.*, 2011) and only one synthetic, the coumarin–naphthoquinone hybrid linked through sulfur spacer attached at 7-position of the coumarin ring and 2-position of the naphthoquinone [2-(7-sulphanyl-4-methyl-coumarinyl)-3-(1-ethoxy)-1,4-naphthoquinone; Ibis & Deniz, 2012]. The title compound (I) is the product of the reaction of 2,3-dicloro-1,4-naphtoquionone with 6-aminocoumarin. The average C—C, C—O, C═O and C—N bond distances are in agreement with those observed in *tert*-butyl *N*-{3-[(3-chloro-1,4-dioxo-1,4-dihydronaphthalen2-yl)amino]propyl}carbamate (Resende & Gomez, 2012). The angle between the naphthoquinone and coumarin planes is 48.99 (6)°. The molecular structure is stabilized by one intramolecular N—H···O hydrogen bond. In the crystal, molecules are linked by strong N—H···O hydrogen bonds into chains with graph-set notation *C*(6) along [101] (Bernstein *et al.*, 1995). The packing also features π–π stacking interactions between naphthoquinone and coumarin rings [centroid–centroid distances = 3.7679 (12) and 3.6180 (13) Å]. The dihedral angle between naphthoquinone and coumarin rings is 48.99 (6)°.

### Experimental

2,3-Dichloro-1,4-naphthoquinone (681 mg, 3 mmol) was added to a solution of 6-aminocoumarin (579.6 mg, 3.6 mmol) in DMF (10 ml). The mixture was stirred at 60–70°C for 72 h. The solvent was evaporated under reduced pressure and the crude product was purified through recrystallization in hexane, resulting in a red solid. Yield: 833.8 mg, 79%. Single crystals suitable for a study of X-ray diffraction of compound (I) were obtained at 4°C by slow evaporation of an acetonitrile–dichloromethane (1:1) solution. m.p. 301°C. Found: C, 64.12; H, 2.91; N, 4.14. Calc. for C_19_H_10_ClNO_4_: C, 64.51; H, 3.42; N, 3.96%. 1H NMR (300 MHz, d6-DMSO): δ 8.16 (d, J = 7.5 Hz, 2H), 8.13 (d, J = 9.6 Hz, 1H), 8.00 (t, J = 7.5 Hz, 1H), 7.94 (t, J = 7.5 Hz, 1H), 7.58–7.52 (m, 2H), 7.48 (d, J = 8.7 Hz, 1H), 6.62 (d, J = 9.6 Hz, 1H). 13 C NMR - APT (d6-DMSO, 75 MHz): δ 180.0, 176.8, 160.0, 150.4, 144.0, 143.3, 135.5, 134.8, 133.4, 131.9, 130.3, 128.0, 126.7, 126.2, 122.7, 118.2, 116.7, 116.0, 114.7. IR (KBr): νC═O (quin.) = 1672, νC═O (ester) = 1720, νC—O (ester) = 1568, 1290, νN—H = 3294, νC—H (arom.) = 3080. UV–Vis [CH_3_CN; λ/nm (log ε)]: 277 (4.10), 333 (3.28), 469 (3.10).

### Refinement

All C-bound H atoms were placed in calculated idealized positions. The N-bound H atom was placed in the calculated idealized position. All H atoms were refined with fixed individual displacement parameters [*U*_iso_(H) = 1.2Ueq using a riding model.

### Crystal data

**Table:**

C_19_H_10_ClNO_4_	*F*(000) = 720
*M**_r_* = 351.73	*D*_x_ = 1.596 Mg m^−^^3^
Monoclinic, *C**c*	Mo *K*α radiation, λ = 0.71073 Å
Hall symbol: C -2yc	Cell parameters from 5237 reflections
*a* = 10.9371 (5) Å	θ = 2.0–29.5°
*b* = 10.4462 (5) Å	µ = 0.29 mm^−^^1^
*c* = 13.5104 (7) Å	*T* = 150 K
β = 108.533 (5)°	Prism, violet
*V* = 1463.53 (12) Å^3^	0.23 × 0.13 × 0.07 mm
*Z* = 4	

### Data collection

**Table:**

Oxford Xcalibur Gemini Ultra diffractometer with Atlas detector	3527 independent reflections
Graphite monochromator	2714 reflections with *I* > 2σ(*I*)
Detector resolution: 10.4186 pixels mm^-1^	*R*_int_ = 0.055
ω scans	θ_max_ = 28.5°, θ_min_ = 2.8°
Absorption correction: multi-scan (*CrysAlis PRO*; Agilent, 2011)	*h* = −14→14
*T*_min_ = 0.947, *T*_max_ = 1	*k* = −13→13
15281 measured reflections	*l* = −18→18

### Refinement

**Table:**

Refinement on *F*^2^	Secondary atom site location: difference Fourier map
Least-squares matrix: full	Hydrogen site location: inferred from neighbouring sites
*R*[*F*^2^ > 2σ(*F*^2^)] = 0.036	H-atom parameters constrained
*wR*(*F*^2^) = 0.065	*w* = 1/[σ^2^(*F*_o_^2^) + (0.029*P*)^2^] where *P* = (*F*_o_^2^ + 2*F*_c_^2^)/3
*S* = 0.91	(Δ/σ)_max_ < 0.001
3527 reflections	Δρ_max_ = 0.23 e Å^−^^3^
226 parameters	Δρ_min_ = −0.21 e Å^−^^3^
2 restraints	Absolute structure: Flack (1983), 1217 Friedel pairs
Primary atom site location: structure-invariant direct methods	Flack parameter: −0.07 (5)

### Special details

**Table:**

Experimental. CrysAlisPro, Oxford Diffraction Ltd., Version 1.171.33.55 (release 05-01-2010 CrysAlis171 .NET) (compiled Jan 5 2010,16:28:46) Empirical absorption correction using spherical harmonics, implemented in SCALE3 ABSPACK scaling algorithm.
Geometry. All e.s.d.'s (except the e.s.d. in the dihedral angle between two l.s. planes) are estimated using the full covariance matrix. The cell e.s.d.'s are taken into account individually in the estimation of e.s.d.'s in distances, angles and torsion angles; correlations between e.s.d.'s in cell parameters are only used when they are defined by crystal symmetry. An approximate (isotropic) treatment of cell e.s.d.'s is used for estimating e.s.d.'s involving l.s. planes.
Refinement. Refinement of *F*^2^ against ALL reflections. The weighted *R*-factor *wR* and goodness of fit *S* are based on *F*^2^, conventional *R*-factors *R* are based on *F*, with *F* set to zero for negative *F*^2^. The threshold expression of *F*^2^ > σ(*F*^2^) is used only for calculating *R*-factors(gt) *etc*. and is not relevant to the choice of reflections for refinement. *R*-factors based on *F*^2^ are statistically about twice as large as those based on *F*, and *R*-factors based on ALL data will be even larger.

### Fractional atomic coordinates and isotropic or equivalent isotropic displacement parameters (Å^2^)

**Table:**

	*x*	*y*	*z*	*U*_iso_*/*U*_eq_	
Cl	0.31971 (5)	0.30204 (5)	0.61162 (4)	0.02411 (12)	
O4	0.57743 (16)	−0.02583 (15)	0.22630 (13)	0.0366 (4)	
O3	0.39509 (13)	0.03816 (14)	0.24617 (11)	0.0246 (4)	
O1	−0.11020 (14)	0.09274 (14)	0.55900 (12)	0.0292 (4)	
O2	0.31861 (14)	0.29316 (15)	0.82346 (11)	0.0279 (4)	
N1	0.07529 (16)	0.14446 (17)	0.48249 (13)	0.0208 (4)	
H1	−0.0048	0.1431	0.4453	0.025*	
C2	0.20172 (19)	0.23128 (19)	0.65296 (15)	0.0182 (5)	
C3	0.09915 (19)	0.17092 (18)	0.58553 (15)	0.0179 (5)	
C14	0.3192 (2)	0.0628 (2)	0.30908 (16)	0.0197 (5)	
C9	0.11248 (18)	0.2050 (2)	0.80240 (15)	0.0182 (4)	
C1	0.2201 (2)	0.2472 (2)	0.76414 (17)	0.0187 (4)	
C11	0.16331 (19)	0.11893 (19)	0.42803 (15)	0.0183 (4)	
C10	−0.00065 (19)	0.1522 (2)	0.73331 (16)	0.0192 (5)	
C13	0.2008 (2)	0.1182 (2)	0.26179 (16)	0.0214 (5)	
H13	0.173	0.1363	0.1891	0.026*	
C4	−0.0132 (2)	0.13481 (18)	0.62218 (16)	0.0191 (5)	
C12	0.1236 (2)	0.14658 (19)	0.32122 (16)	0.0211 (5)	
H12	0.042	0.1856	0.2895	0.025*	
C15	0.36188 (19)	0.03463 (19)	0.41469 (17)	0.0195 (5)	
C16	0.28168 (19)	0.06194 (19)	0.47438 (16)	0.0202 (5)	
H16	0.3085	0.0414	0.5466	0.024*	
C19	0.5182 (2)	−0.0122 (2)	0.28635 (19)	0.0269 (5)	
C18	0.5609 (2)	−0.0425 (2)	0.39665 (18)	0.0258 (5)	
H18	0.6438	−0.0795	0.4265	0.031*	
C8	0.1236 (2)	0.2211 (2)	0.90669 (17)	0.0240 (5)	
H8	0.1993	0.258	0.9536	0.029*	
C5	−0.1001 (2)	0.1138 (2)	0.76989 (17)	0.0243 (5)	
H5	−0.1765	0.0775	0.7235	0.029*	
C6	−0.0869 (2)	0.1289 (2)	0.87452 (18)	0.0265 (5)	
H6	−0.1543	0.1017	0.9	0.032*	
C7	0.0230 (2)	0.1829 (2)	0.94223 (17)	0.0264 (5)	
H7	0.0301	0.1942	1.0136	0.032*	
C17	0.4889 (2)	−0.0209 (2)	0.45813 (18)	0.0249 (5)	
H17	0.5209	−0.0419	0.5302	0.03*	

### Atomic displacement parameters (Å^2^)

**Table:**

	*U*^11^	*U*^22^	*U*^33^	*U*^12^	*U*^13^	*U*^23^
Cl	0.0214 (2)	0.0303 (3)	0.0225 (2)	−0.0092 (3)	0.0095 (2)	−0.0010 (3)
O4	0.0313 (10)	0.0461 (11)	0.0399 (10)	0.0050 (8)	0.0221 (8)	−0.0034 (8)
O3	0.0222 (8)	0.0287 (9)	0.0246 (9)	0.0013 (7)	0.0101 (7)	−0.0018 (6)
O1	0.0234 (9)	0.0360 (10)	0.0286 (9)	−0.0082 (7)	0.0091 (7)	−0.0020 (7)
O2	0.0187 (8)	0.0391 (10)	0.0252 (8)	−0.0062 (7)	0.0060 (7)	−0.0032 (7)
N1	0.0147 (9)	0.0263 (10)	0.0214 (10)	−0.0009 (7)	0.0055 (8)	−0.0011 (7)
C2	0.0175 (11)	0.0185 (12)	0.0225 (12)	−0.0010 (8)	0.0120 (9)	0.0029 (8)
C3	0.0188 (11)	0.0156 (11)	0.0196 (11)	0.0036 (9)	0.0065 (9)	0.0045 (8)
C14	0.0191 (11)	0.0205 (11)	0.0206 (11)	−0.0041 (9)	0.0079 (9)	−0.0025 (9)
C9	0.0186 (11)	0.0159 (10)	0.0226 (11)	0.0040 (9)	0.0099 (9)	0.0033 (9)
C1	0.0152 (10)	0.0172 (11)	0.0231 (11)	0.0033 (8)	0.0053 (9)	0.0011 (8)
C11	0.0190 (10)	0.0176 (11)	0.0199 (11)	−0.0042 (9)	0.0085 (9)	−0.0047 (9)
C10	0.0170 (11)	0.0149 (11)	0.0279 (12)	0.0057 (8)	0.0103 (9)	0.0041 (9)
C13	0.0230 (11)	0.0235 (12)	0.0165 (11)	−0.0028 (9)	0.0045 (9)	−0.0022 (9)
C4	0.0177 (11)	0.0140 (11)	0.0253 (12)	0.0014 (9)	0.0066 (10)	0.0015 (9)
C12	0.0166 (10)	0.0204 (12)	0.0235 (12)	−0.0001 (9)	0.0027 (9)	−0.0007 (9)
C15	0.0160 (11)	0.0161 (11)	0.0261 (12)	−0.0014 (8)	0.0065 (9)	−0.0005 (8)
C16	0.0210 (11)	0.0207 (12)	0.0186 (11)	0.0007 (9)	0.0060 (9)	−0.0004 (8)
C19	0.0231 (12)	0.0208 (12)	0.0390 (14)	0.0007 (10)	0.0127 (11)	−0.0014 (10)
C18	0.0183 (12)	0.0252 (13)	0.0328 (13)	0.0053 (9)	0.0067 (10)	0.0037 (10)
C8	0.0224 (12)	0.0268 (13)	0.0231 (12)	0.0055 (9)	0.0076 (10)	0.0029 (9)
C5	0.0201 (11)	0.0201 (12)	0.0360 (14)	0.0017 (9)	0.0135 (10)	0.0020 (10)
C6	0.0232 (12)	0.0298 (14)	0.0338 (14)	0.0067 (10)	0.0194 (11)	0.0070 (10)
C7	0.0270 (12)	0.0339 (15)	0.0230 (12)	0.0098 (11)	0.0146 (10)	0.0059 (10)
C17	0.0245 (13)	0.0222 (12)	0.0262 (12)	0.0014 (10)	0.0054 (11)	0.0028 (10)

### Geometric parameters (Å, º)

**Table:**

Cl—C2	1.7268 (19)	C10—C5	1.389 (3)
O4—C19	1.197 (2)	C10—C4	1.475 (3)
O3—C19	1.385 (3)	C13—C12	1.370 (3)
O3—C14	1.387 (2)	C13—H13	0.95
O1—C4	1.213 (2)	C12—H12	0.95
O2—C1	1.219 (3)	C15—C16	1.397 (3)
N1—C3	1.361 (2)	C15—C17	1.446 (3)
N1—C11	1.411 (2)	C16—H16	0.95
N1—H1	0.86	C19—C18	1.448 (3)
C2—C3	1.355 (3)	C18—C17	1.333 (3)
C2—C1	1.460 (3)	C18—H18	0.95
C3—C4	1.511 (3)	C8—C7	1.391 (3)
C14—C13	1.376 (3)	C8—H8	0.95
C14—C15	1.385 (3)	C5—C6	1.384 (3)
C9—C8	1.386 (3)	C5—H5	0.95
C9—C10	1.404 (3)	C6—C7	1.379 (3)
C9—C1	1.494 (3)	C6—H6	0.95
C11—C16	1.381 (3)	C7—H7	0.95
C11—C12	1.399 (3)	C17—H17	0.95
			
C19—O3—C14	121.75 (17)	C13—C12—C11	120.85 (19)
C3—N1—C11	129.15 (18)	C13—C12—H12	119.6
C3—N1—H1	115.4	C11—C12—H12	119.6
C11—N1—H1	115.4	C14—C15—C16	118.94 (18)
C3—C2—C1	123.94 (18)	C14—C15—C17	117.96 (18)
C3—C2—Cl	121.72 (15)	C16—C15—C17	123.1 (2)
C1—C2—Cl	114.29 (15)	C11—C16—C15	119.77 (19)
C2—C3—N1	129.12 (18)	C11—C16—H16	120.1
C2—C3—C4	118.78 (17)	C15—C16—H16	120.1
N1—C3—C4	111.90 (17)	O4—C19—O3	116.6 (2)
C13—C14—C15	121.78 (18)	O4—C19—C18	127.2 (2)
C13—C14—O3	116.84 (18)	O3—C19—C18	116.22 (18)
C15—C14—O3	121.37 (18)	C17—C18—C19	122.9 (2)
C8—C9—C10	119.83 (18)	C17—C18—H18	118.6
C8—C9—C1	119.46 (18)	C19—C18—H18	118.6
C10—C9—C1	120.69 (17)	C9—C8—C7	119.5 (2)
O2—C1—C2	121.67 (19)	C9—C8—H8	120.2
O2—C1—C9	121.19 (19)	C7—C8—H8	120.2
C2—C1—C9	117.14 (18)	C6—C5—C10	119.4 (2)
C16—C11—C12	119.73 (18)	C6—C5—H5	120.3
C16—C11—N1	122.81 (18)	C10—C5—H5	120.3
C12—C11—N1	117.36 (18)	C7—C6—C5	120.7 (2)
C5—C10—C9	120.07 (19)	C7—C6—H6	119.7
C5—C10—C4	119.79 (19)	C5—C6—H6	119.7
C9—C10—C4	120.13 (18)	C6—C7—C8	120.4 (2)
C12—C13—C14	118.92 (19)	C6—C7—H7	119.8
C12—C13—H13	120.5	C8—C7—H7	119.8
C14—C13—H13	120.5	C18—C17—C15	119.8 (2)
O1—C4—C10	122.55 (18)	C18—C17—H17	120.1
O1—C4—C3	118.71 (18)	C15—C17—H17	120.1
C10—C4—C3	118.74 (18)		
			
C1—C2—C3—N1	−176.13 (19)	N1—C3—C4—O1	−2.7 (3)
Cl—C2—C3—N1	6.5 (3)	C2—C3—C4—C10	−7.3 (3)
C1—C2—C3—C4	9.5 (3)	N1—C3—C4—C10	177.40 (17)
Cl—C2—C3—C4	−167.80 (14)	C14—C13—C12—C11	−0.6 (3)
C11—N1—C3—C2	30.9 (3)	C16—C11—C12—C13	−0.2 (3)
C11—N1—C3—C4	−154.41 (19)	N1—C11—C12—C13	−176.49 (18)
C19—O3—C14—C13	177.18 (19)	C13—C14—C15—C16	0.7 (3)
C19—O3—C14—C15	−1.9 (3)	O3—C14—C15—C16	179.77 (18)
C3—C2—C1—O2	174.1 (2)	C13—C14—C15—C17	−178.7 (2)
Cl—C2—C1—O2	−8.4 (3)	O3—C14—C15—C17	0.4 (3)
C3—C2—C1—C9	−6.0 (3)	C12—C11—C16—C15	1.2 (3)
Cl—C2—C1—C9	171.51 (14)	N1—C11—C16—C15	177.35 (18)
C8—C9—C1—O2	1.6 (3)	C14—C15—C16—C11	−1.5 (3)
C10—C9—C1—O2	179.98 (19)	C17—C15—C16—C11	177.8 (2)
C8—C9—C1—C2	−178.30 (18)	C14—O3—C19—O4	−177.49 (19)
C10—C9—C1—C2	0.1 (3)	C14—O3—C19—C18	2.5 (3)
C3—N1—C11—C16	29.8 (3)	O4—C19—C18—C17	178.2 (2)
C3—N1—C11—C12	−154.0 (2)	O3—C19—C18—C17	−1.8 (3)
C8—C9—C10—C5	−1.4 (3)	C10—C9—C8—C7	1.0 (3)
C1—C9—C10—C5	−179.75 (18)	C1—C9—C8—C7	179.39 (19)
C8—C9—C10—C4	179.97 (18)	C9—C10—C5—C6	0.5 (3)
C1—C9—C10—C4	1.6 (3)	C4—C10—C5—C6	179.15 (19)
C15—C14—C13—C12	0.4 (3)	C10—C5—C6—C7	0.8 (3)
O3—C14—C13—C12	−178.74 (18)	C5—C6—C7—C8	−1.2 (3)
C5—C10—C4—O1	3.3 (3)	C9—C8—C7—C6	0.3 (3)
C9—C10—C4—O1	−178.1 (2)	C19—C18—C17—C15	0.4 (3)
C5—C10—C4—C3	−176.81 (17)	C14—C15—C17—C18	0.4 (3)
C9—C10—C4—C3	1.9 (3)	C16—C15—C17—C18	−179.0 (2)
C2—C3—C4—O1	172.59 (19)		

### Hydrogen-bond geometry (Å, º)

**Table:**

*D*—H···*A*	*D*—H	H···*A*	*D*···*A*	*D*—H···*A*
N1—H1···O2^i^	0.86	2.21	3.015 (2)	157

Symmetry code: (i) *x*−1/2, −*y*+1/2, *z*−1/2.
